# A Rational Insight into the Effect of Dimethyl Sulfoxide on TNF-α Activity

**DOI:** 10.3390/ijms21249450

**Published:** 2020-12-11

**Authors:** Nasir Javaid, Mahesh Chandra Patra, Hana Seo, Farzana Yasmeen, Sangdun Choi

**Affiliations:** 1 Department of Molecular Science and Technology, Ajou University, Suwon 16499, Korea; nasirjavaid@ajou.ac.kr (N.J.); ml2mahesh@ajou.ac.kr (M.C.P.); cos159159@ajou.ac.kr (H.S.); farzana892019@ajou.ac.kr (F.Y.); 2 S&K Therapeutics, Woncheon Hall 135, Ajou University, Suwon 16499, Korea

**Keywords:** tumor necrosis factor-alpha, dimethyl sulfoxide, trimerization, fibroblast

## Abstract

Direct inhibition of tumor necrosis factor-alpha (TNF-α) action is considered a promising way to prevent or treat TNF-α-associated diseases. The trimeric form of TNF-α binds to its receptor (TNFR) and activates the downstream signaling pathway. The interaction of TNF-α with molecular-grade dimethyl sulfoxide (DMSO) in an equal volumetric ratio renders TNF-α inert, in this state, TNF-α fails to activate TNFR. Here, we aimed to examine the inhibition of TNF-α function by various concentrations of DMSO. Its higher concentration led to stronger attenuation of TNF-α-induced cytokine secretion by fibroblasts, and of their death. We found that this inhibition was mediated by a perturbation in the formation of the functional TNF-α trimer. Molecular dynamics simulations revealed a transient interaction between DMSO molecules and the central hydrophobic cavity of the TNF-α homodimer, indicating that a brief interaction of DMSO with the TNF-α homodimer may disrupt the formation of the functional homotrimer. We also found that the sensitizing effect of actinomycin D on TNF-α-induced cell death depends upon the timing of these treatments and on the cell type. This study will help to select an appropriate concentration of DMSO as a working solvent for the screening of water-insoluble TNF-α inhibitors.

## 1. Introduction

Multiple proinflammatory cytokines are members of the tumor necrosis factor (TNF) superfamily and are involved in several physiological processes, including tumor prevention and host protection [1,2]. A slight dysregulation of TNF subtypes (especially TNF-α) may lead to several pathological conditions such as psoriasis, rheumatoid arthritis, [3], and inflammatory bowel disease [4]. Continuous TNF-α production is also associated with cancer [5], depression [6], and Alzheimer’s disease [7]. Accordingly, multiple therapeutic modulators are being designed to disrupt TNF signaling pathways [8].

TNF is a homo-trimeric cellular cytokine that is converted into a soluble form via proteolytic cleavage of the transmembrane form. Monomeric units of the bell-shaped trimeric form are held together via noncovalent interactions between them [9,10]. The trimeric form of both soluble and transmembrane TNF interacts with TNF receptor I and/or II to bring some conformational changes to their complex, which leads to the activation of downstream signaling pathways [11,12,13,14]. At physiological concentrations (pg/mL–ng/mL), soluble TNF is unstable in serum or buffer and converts itself into a reversible inactive monomeric form [15,16,17]. In addition, other factors can affect the stability of trimers, for example, pH (less than 5 or more than 9) and temperature [18]. The oligomeric form of TNF-α is also disturbed by certain solvents, such as dimethyl sulfoxide (DMSO) [16,19].

Most drug-like molecules have a medium size and contain 10–50 atoms in addition to hydrogen. They mainly constitute interlinked aromatic cores along with various substituents composed of heteroatoms such as P, N, S, O, and X (e.g., F, Cl). Because of this structure, they are highly polarizable and conformationally flexible. This flexibility contributes to the reactivity, solvation, and formation of crystal polymorphs [20]. The presence of heteroatoms along with high polarizability allows them to interact with polar solvents by hydrogen bonding, protonation, or specific solvation [21,22,23]. The solubility of drug-like molecules in aqueous solutions poses a major obstacle in the process of drug discovery [24]. Poorly soluble molecules cause problems during in vitro and in vivo drug discovery assays and pose a risk of failure during the developmental process [25,26]. Such molecules are mostly dissolved in DMSO for initial screening because DMSO is considered the most powerful organic solvent able to dissolve a variety of organic substances with a higher dielectric constant [27]. DMSO also has low environmental toxicity and low toxicity via every administration route (such as inhalation, oral, and dermal) [28,29]. Nonetheless, the presence of DMSO in a solution of molecules may affect the process of screening of the molecules targeting TNF-α [16,19]. Recently, a molecule has been reported to inhibit TNF-α signaling by stabilizing the asymmetric conformation of its trimer. The higher potency of the molecule overcomes the tendency of DMSO to disrupt the TNF-α trimer [30]. However, the inhibitory mechanism could not be the same for all the small molecules. The molecules disrupting the trimeric form of TNF-α might not be interpreted well, as their solvent (i.e., DMSO) would also have its own disrupting effect. Hence, it is necessary to analyze the concentration of DMSO, which has minimum effect on disrupting the TNF-α trimer despite the discovery of molecules inhibiting the production of TNF-α [31,32,33] or TNF-α itself [34,35,36,37].

Here, we aimed to explore the effects of different concentrations of DMSO on TNF-α-mediated signaling pathways at the molecular level. We determined the concentration has a minimal effect and is well suited for the screening of compounds. We also predicted the inhibitory mechanism of action of DMSO on TNF-α by in vitro assays and in silico simulations.

## 2. Results

### 2.1. DMSO Inhibits TNF-α-Induced Cytokine Secretion and Cell Proliferation

**Figure 1:**
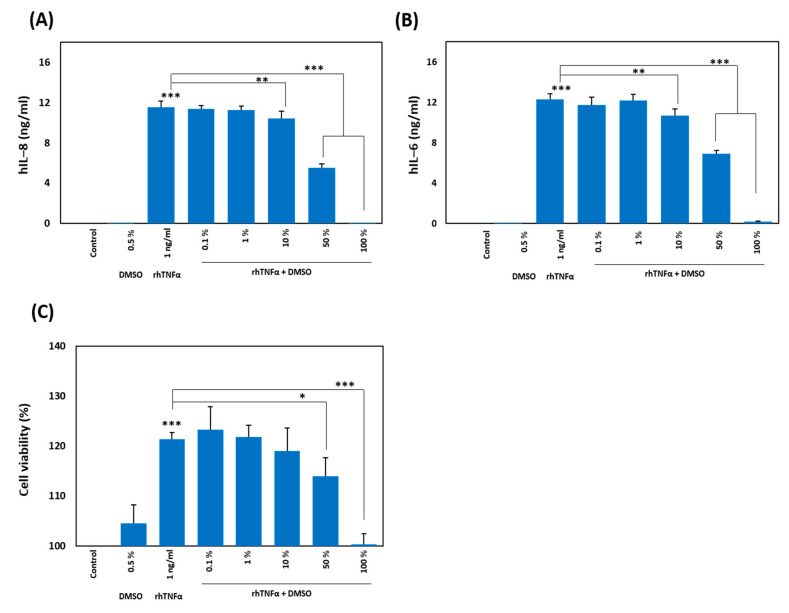
Tumor necrosis factor-alpha (TNF-α)-induced cytokine secretion and proliferation. (**A**,**B**) Human dermal fibroblasts (HDFs) (10^4^/well) were treated for 24 h with either recombinant human TNF-α (rhTNF-α; 1 ng/mL) only or the indicated concentrations of dimethyl sulfoxide (DMSO) preincubated for 1 h with rhTNF-α. The supernatant was processed for the ELISA to evaluate the secretion level of human interleukin (hIL)-8 (**A**) and hIL-6 (**B**). The data were normalized to the control. (**C**) The HDFs were treated as mentioned above, and cell viability was evaluated by the 1-(4,5-dimethylthiazol-2-yl)-3,5-diphenylformazan (MTT) assay. All experiments were independently conducted four times, and differences between means of the experiments (±SEM) were evaluated by a two-tailed paired Student’s *t*-test (* *p* < 0.05, ** *p* < 0.01, *** *p* < 0.001).

TNF-α is produced by various immune cells and activates nuclear factor κ-light-chain enhancer of activated B cells (NF-κB) and mitogen-activated protein kinases (MAPKs) via its receptors (TNFR1 and TNFR2), thereby leading to the production of multiple inflammatory cytokines [38,39,40,41]. To elucidate the impact of DMSO on TNF-α at the molecular level, we prepared different solutions of DMSO in water: 0.1%, 1%, 10%, 50%, and 100%. Each sample was separately incubated with recombinant human TNF-α (rhTNF-α; 1 ng/mL) in an equal volumetric ratio (1:1, *v*/*v*) for 1 h at room temperature. The mixture was then incubated with human dermal fibroblasts (HDFs) for 24 h, which converted the final concentration of DMSO to 0.0005%, 0.005%, 0.05%, 0.25%, and 0.5%, respectively. It was followed by collection of the supernatant and an ELISA to measure the secretion level of human interleukin-8 (hIL-8) and hIL-6. We observed a decrease in the secretion of hIL-8 (Figure 1A) and hIL-6 (Figure 1B) with the increasing concentration of DMSO in solution, that is, the 100% DMSO solution completely inhibited the secretion of both cytokines, while this effect was nonsignificant at 0.1% and 1%. It has been reported that a final concentration of DMSO of 1% or more can affect multiple cellular processes, so we made sure in our study that the final concentration did not exceed that limit [42,43,44]. TNF-α has been reported to increase the proliferation of target cells by activating cell cycle regulators (c-Myc and Ras) or cell survival molecules (MAPKs, NF-κB, and PI3K–Akt), or by decreasing the expression of Cdk inhibitors [45,46]. Hence, we examined this phenomenon in HDFs by activating them with rhTNF-α (1 ng/mL) for 24 h. To elucidate the influence of DMSO on TNF-α-induced proliferation, we treated HDFs with a mixture of rhTNF-α with a solution of DMSO at various concentrations (as mentioned above; each preincubated for 1 h) and performed a cell viability assay after 24 h. The increase in TNF-α-induced proliferation was attenuated with an increase in the concentration of DMSO, and there was complete inhibition with a solution containing 100% DMSO (Figure 1C).

### 2.2. DMSO Inhibits TNF-α-Induced Cell Death

**Figure 2:**
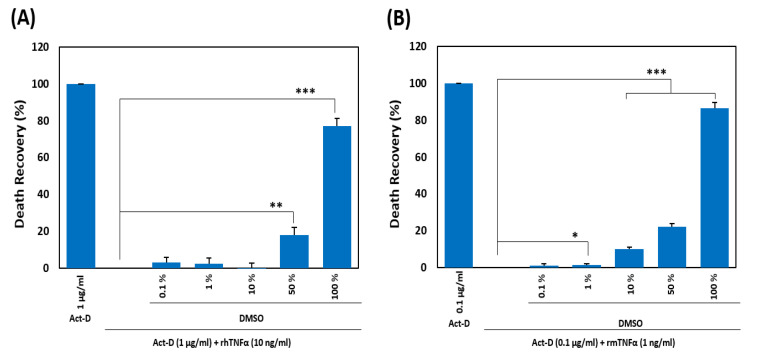
Inhibition of tumor necrosis factor-alpha (TNF-α)-induced cell death. (**A**,**B**) A comparison of the death recovery rate (%) between HDFs (10^4^/well) (**A**) and L929 cells (1.5 × 10^4^/well) (**B**) was carried out by treating them for 24 h with either TNF-α only or the indicated concentrations of DMSO preincubated for 1 h with rhTNF-α. The cell viability was measured by a colorimetric MTT assay. All experiments were independently conducted four times and means ± SEM of the independent experiments were subjected to a two-tailed paired Student’s *t*-test (* *p* < 0.05, ** *p* < 0.01, *** *p* < 0.001). Act-D = actinomycin D.

Along with cell proliferation, TNF-α causes inflammatory cell death by activating associated death signaling pathways [47]. To evaluate the preventive effect of DMSO on TNF-α-induced apoptosis, we preincubated rhTNF-α with a solution of DMSO in water at different concentrations (0.1%, 1%, 10%, 50%, and 100%) and applied it to actinomycin D (act-D)-sensitized HDFs at a final concentration of DMSO of 0.0005%, 0.005%, 0.05%, 0.25%, and 0.5%, respectively. We observed significant inhibition of TNF-α-induced apoptosis with the increasing concentration of DMSO, with maximum inhibition seen with the 100% DMSO solution (Figure 2A). To reproduce the effect in mouse cells, the preincubated mixtures of different concentrations of DMSO (as mentioned above) with recombinant mouse TNF-α (rmTNF-α) were added to the act-D-sensitized L929 cell line. As expected, DMSO also inhibited the rmTNF-α-induced death of the murine fibroblast line in a dose-dependent manner (Figure 2B).

### 2.3. DMSO Suppresses Multiple TNF-α-Mediated Signaling Pathways

**Figure 3:**
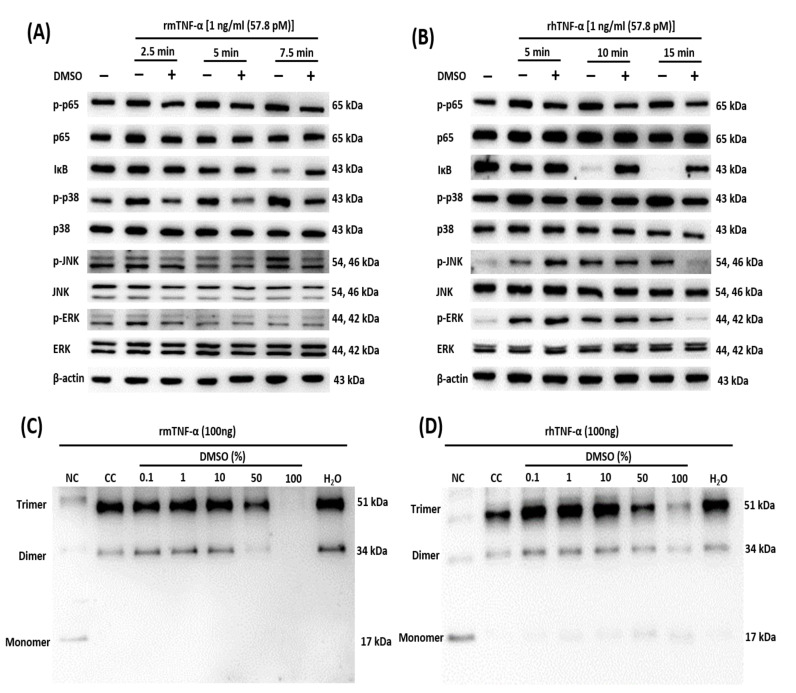
Inhibition of TNF-α-dependent signaling pathways by disruption of the oligomeric form. (**A**,**B**) The inhibition of NF-κB and MAPK-signaling pathways by DMSO (100%) was evaluated by treating L929 cells (**A**) and HDFs (**B**) at the indicated time points with either TNF-α only or DMSO preincubated for 1 h with rhTNF-α. (**C**,**D**) Disruption of homotrimerization of recombinant mouse TNF-α (rmTNF-α (**C**)) and recombinant human TNF-α (rhTNF-α (**D**)) after the treatment with DMSO was evaluated. Human and mouse TNF-α were incubated with DMSO, chemically cross-linked, and subjected to Western blotting. NC = non-cross-linked control (no cross-linker, no inhibitor); CC = cross-linked control (no inhibitor). The blots were visualized on a ChemiDoc™ Touch Imaging System (Bio-Rad Laboratories).

Multiple signaling pathways, including NF-κB and MAPK, are activated by the stimulation of TNFR1 with its ligand, TNF-α [48]. For the NF-κB and MAPK pathways, we treated L929 cells and HDFs at different time points with either TNF-α only or a preincubated mixture of TNF-α with DMSO (100%). We performed a Western blot analysis to examine the effect of DMSO on the TNF-α-induced activation of NF-κB (phosphorylation of p65 and degradation of IκB) and MAPKs (phosphorylation of p38, JNK, and ERK). We observed the inhibition of these pathways with DMSO in L929 cells (Figure 3A) and HDFs (Figure 3B). DMSO did not affect the amounts of total p65, p38, JNK, and ERK. For the assay of the caspase pathway, we treated sensitized L929 cells and HDFs with either TNF-α only or the preincubated mixture of TNF-α with DMSO (100%).

### 2.4. DMSO Prevents Oligomerization of TNF-α

After we confirmed the inhibitory impact of DMSO on the TNF-α signaling pathway, the DMSO mechanism of action was evaluated in a protein cross-linking interaction assay with subsequent Western blot analyses. A perturbation in the homotrimeric form of TNF-α interferes with its functionality and causes the inhibition of cytokine secretion and cell death [37,49,50]. To this end, rhTNF-α and rmTNF-α were separately preincubated with DMSO, chemically cross-linked, and analyzed by Western blotting. As expected, DMSO inhibited the formation of functional homotrimers in both rmTNF-α (Figure 3C) and rhTNF-α (Figure 3D) in a concentration-dependent manner. In the case of rhTNF-α, we unexpectedly observed a stronger formation of the trimer at ≤10% DMSO, as compared with the cross-linked control (CC), and the same was the case for water only (H_2_O; Figure 3D). The mechanism underlying this species-specific action of water on TNF-α needs to be confirmed.

### 2.5. The DMSO Molecules Engage in a Transient Interaction within the Hydrophobic Cavity of the TNF Homodimer

**Figure 4:**
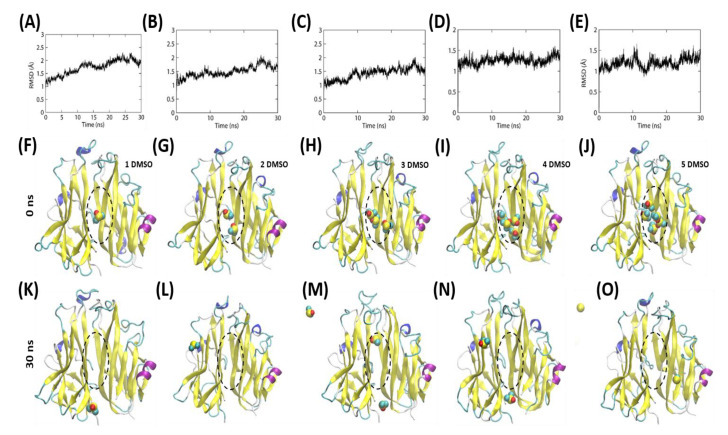
Molecular dynamics (MD) simulation of TNF and different numbers of DMSO molecules in its ligand-binding cavity. (**A**–**E**) Root mean square deviation (RMSD) of the backbone atoms. (**F**–**J**) The starting conformations of the TNF homodimer with different numbers of DMSO molecules in the ligand-binding site. (**K**–**O**) The final snapshot structure of the TNF–DMSO complex after a 30 ns MD simulation.

To understand the possible mode of interaction between DMSO and TNF-α, we performed molecular dynamics (MD) simulations by placing different numbers of DMSO molecules inside the hydrophobic cavity of the TNF homodimer. The simulations were performed for 30 ns (Figure 4A–E), and the simulations were continued until at least one DMSO molecule was present inside the hydrophobic cavity of the TNF homodimer. We observed that under the dynamic conditions, the DMSO molecules quickly oscillated in the binding cavity of TNF and dissociated (entered the solvent) after a brief interaction (Figure 4F–O). Visual observation of the DMSO motion along the trajectory indicated that the TNF could contain at least one DMSO molecule in the ligand-binding site. Nonetheless, depending on the initial orientation of the molecular placement, the DMSO molecules gradually tend to dissociate from the TNF cavity (Supplementary Movies S1–S5). This indicates that the DMSO molecules could engage in a transient interaction and prevent the formation of a TNF homotrimer that may last for a short period.

### 2.6. Pretreatment with TNF-α Weakens the Sensitization by Act-D in a Timing-Dependent Manner

**Figure 5:**
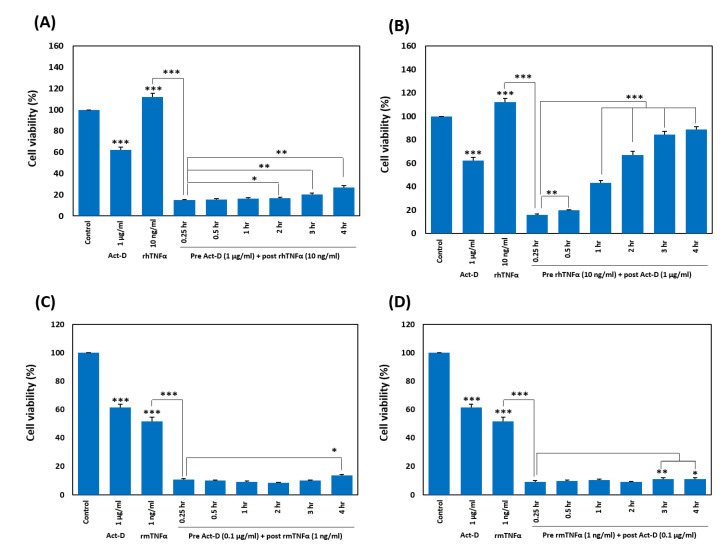
Timing-dependent attenuation of sensitization by act-D. (**A**,**C**) Both HDFs (**A**) and L929 cells (**C**) were pretreated with act-D, followed by treatment with respective TNF-α at the indicated time points. After 24 h, cell viability was measured by the MTT assay. (**B**,**D**) Both HDFs (**B**) and L929 cells (**D**) were pretreated with respective TNF-α, followed by treatment with act-D at the indicated time points. After 24 h, cell viability was measured by the MTT assay. All experiments were independently conducted four times and means ± SEM of the independent experiments were subjected to a two-tailed paired Student’s *t*-test (* *p* < 0.05, ** *p* < 0.01, *** *p* < 0.001).

While optimizing the TNF-α-induced cell death, we pretreated HDFs and L929 cells with act-D, followed by treatment with TNF-α at different time points (0.25, 0.5, 1, 2, 3, and 4 h). After overnight incubation, we quantitated the effect by calculating cell viability in the MTT assay and found a gradual decrease in TNF-α-induced cell death with the increasing time gap between the two treatments. This phenomenon was more prominent in HDFs (Figure 5A) than in L929 cells (Figure 5C). We also analyzed the impact by reversing the treatment sequence of TNF-α and act-D, that is, pretreatment with TNF-α and post-treatment with act-D at the time points mentioned above. We noted a similar pattern but with a more prominent reduction in TNF-α-induced deaths of HDFs as compared with the first combination (Figure 5B). By contrast, we did not see such a pattern in L929 cells up to 4 h (Figure 5D).

## 3. Discussion

In drug discovery, solubility has a key effect on bioassays, in vivo formulation, and intestinal absorption. The solubility of a molecule depends upon the solution conditions and the structure of the molecule. The conditions of the solution are influenced by cosolvents, pH, additives, time, temperature, or ionic strength. The structure of the molecule determines hydrogen bonding, lipophilicity, crystal energy, molecular volume, and ionizability. DMSO is a polar organic aprotic molecule that is capable of dissolving diverse drug-like compounds because of its amphipathic nature [51]. Owing to its water displacement and membrane penetration properties, DMSO is also commonly used as a cryopreservative. It is also being employed as a vehicle for various in vitro and in vivo studies [52,53,54]. Along with good solubility properties, some studies have revealed a toxic effect of DMSO [55,56].

TNF-α is a strong proinflammatory cytokine with multiple effects on different cell types and plays a major role in inflammatory diseases, such as rheumatoid arthritis [57,58]. It is expressed in a precursor transmembrane form (26 kDa) on the surface of lymphocytes, activated macrophages, and some other cell types [59,60,61]. After being cleaved by a TNF-α-converting enzyme (TACE), the soluble form (17 kDa) is secreted to perform its biological function via type 1 and 2 TNF receptors (TNF-R1 and TNF-R2, respectively) [62,63]. Both soluble and transmembrane forms of TNF-α oligomerize to form functional homotrimers [64]. This oligomeric version of TNF-α has been reported to be disturbed by solvents of drug-like molecules such as DMSO [16,19].

TNF-α has been found to induce the production of multiple inflammatory cytokines [38,39,40,41] and to increase proliferation of target cells [45,46], and the death of some cells [47]. In our study, we examined the impact of DMSO on these TNF-α-induced processes and found that the extent of their attenuation is directly related to the concentration of DMSO used. DMSO at 0.1% and 1% did not inhibit the TNF-α-mediated molecular pathways, whereas higher concentrations affected these processes in a dose-dependent manner. Later, we confirmed the mechanism of inhibition of these processes and found that it is mediated by the perturbation of TNF-α oligomer confirmation. Previous studies have also documented the disruption of the TNF-α trimer by DMSO. It has been reported that the TNF-α trimer can be disturbed by 5–10% DMSO; for example, 10% DMSO could yield 40% monomer in the solution [16]. In another study, more than 90% of TNF-α was converted into the monomeric form by 25% DMSO [19]. Our MD simulations suggest that DMSO molecules can interact with the hydrophobic cavity of TNF; although for a brief period, this transient interaction might be sufficient to hinder the formation and function of the TNF trimer in a physiological environment. Hence, DMSO has a significant impact on the functionality of proteins of hydrophobic nature, such as TNF-α. This drawback could be overcome if alternative organic solvents are used with drug-like compounds [65].

Activation of the TNF-α signaling pathway leads to either cell death or cell proliferation and survival [41]. The triggering of TNF-α receptors activates NF-κB–stimulating complex I and caspase-8-activating complex II [66]. Cells with stronger activation of NF-κB show survival, while those with weaker activation of NF-κB undergo death [67,68]. To examine TNF-α-induced death in cells with stronger NF-κB activation, the NF-κB pathway is inhibited by blocking protein or RNA synthesis by means of cycloheximide or act-D, respectively [69,70]. It has been found that some cells, including PC12 cells, cortical neurons [71], and mouse hepatocytes [72] require the inhibition of the NF-κB pathway for cell death. The mouse fibroblast cell line, L929, is also reported to require inhibition of the NF-κB pathway by transcriptional arrest to allow TNF-α-induced cell death [50,73]. In our study, we detected the death of the L929 cell line after treatment with rmTNF-α alone. In our study, HDFs showed proliferation after treatment with rhTNF-α alone. By contrast, upon sensitizing HDFs and L929 cells with act-D, we observed significant cell death during the treatment with TNF-α after 15 min of sensitization. This effect became weaker with the increasing time gap between act-D and TNF-α with a prominent reduction in the case of HDFs, as compared with L929 cells. This finding indicates that the effect of transcriptional arrest by act-D is not permanent but gradually decreases with time. By reversing the treatment sequence for TNF-α and act-D, we achieved a more prominent reduction in TNF-α-induced deaths of HDFs by increasing the time gaps between the treatments. This result indicates that earlier inhibition of cell survival pathways is required to make the cell death response more prominent in cells with stronger NF-κB activation. Moreover, the absence of timing phenomena in the TNF-α-mediated reduction in the death of L929 cells means that these phenomena are dependent on the cell type and are absent in cells with weaker activation of cell survival or proliferative pathways.

## 4. Methods

### 4.1. Cell Lines and Reagents

HDFs (American Type Culture Collection (ATCC), Manassas, VA, USA) and L929 cells (ATCC, Manassas, VA, USA) were grown in high-glucose DMEM supplemented with 10% of fetal bovine serum (FBS) (Thermo Fisher Scientific, Inc., Waltham, MA, USA), 1% of a penicillin/streptomycin solution, and 0.2% of Normocin solution (InvivoGen, San Diego, CA, USA). All the cells were incubated in a humidified atmosphere containing 5% CO_2_ at 37 °C (Thermo Fisher Scientific, Inc.). DMSO (Sigma-Aldrich Corp., St. Louis, MO, USA.), rmTNF-α (Miltenyi Biotec, Auburn, CA, USA), rhTNF-α (Miltenyi Biotec, Auburn, CA, USA), act-D (Thermo Fisher Scientific, Inc.), and BS3 (Thermo Fisher Scientific, Inc.) were purchased from the companies mentioned in the parenthesis.

### 4.2. The Cell Viability Assay

An MTT assay (Sigma-Aldrich) was performed to measure cell viability. Briefly, HDFs and L929 cells were seeded in 96-well plates (BD Biosciences, San Jose, CA, USA) at a density of 10^4^/well and 1.5 × 10^4^/well, respectively, and grown overnight. After the completion of treatment, the medium was replaced with a 10% MTT solution (100 μL/well), and the cells were incubated for 3 h, followed by replacement of the solution and incubation with DMSO (100 μL/well) for 30 min. Finally, absorbance was measured at 540 nm on a spectrophotometer (Molecular Devices, Silicon Valley, CA, USA).

### 4.3. The Death Recovery Assay

HDFs and L929 cells were seeded at appropriate densities (mentioned above) in 96-well plates and grown overnight under suitable conditions. The next day, HDFs and L929 cells were pretreated with 1 and 0.1 µg/mL act-D, respectively, and incubated for 15 min. After that, a mixture of 10 ng/mL rhTNF-α with different concentrations of DMSO (0.1–100%) or 1 ng/mL rmTNF-α with different concentrations of DMSO (0.1–100%) (each preincubated for 1 h) was applied to the HDFs and L929 cells, respectively. The cells were incubated for 24 h post-treatment, and survival was measured by the MTT assay. Cell viability was calculated with reference to the no-treatment control. The obtained values were normalized to the act-D treatment group and were then used to calculate the death attenuation rate (%) using the following formula:

Death attenuation (%) = 100 − (((100-sample value)/(100-ActD and TNFα cotreatment value)) ×100)

### 4.4. Western Blot Analyses

The mammalian protein extraction reagent, M-PER (Thermo Fisher Scientific, Inc.), was used to isolate total protein from treated cells. The Bicinchoninic Acid (BCA) Assay Kit (Sigma-Aldrich) was utilized to measure the protein concentration. A Mini-PROTEAN Tetra Cell and Mini Trans-Blot Electrophoretic Transfer Cell System (Bio-Rad Laboratories, Hercules, CA, USA) were used for protein separation and transfer, respectively. The membranes were immunoblotted with primary antibodies, including those against phospho- (p-)p65, p65, p-JNK, JNK, ERK, p-p38, p38, IKB, (Cell Signaling Technology Inc., Danvers, MA, USA), p-ERK, and β-actin (Santa Cruz Biotechnology Inc., Dallas, TX, USA) with gentle shaking at 4 °C overnight. The next day, membranes were rigorously washed with PBS containing 0.05% of Tween 20 and incubated at room temperature with a peroxidase-conjugated anti-rabbit or anti-mouse IgG antibody for 2 h. The SuperSignal West Pico ECL solution (Thermo Fisher Scientific, Inc.) was applied to detect proteins, and visualization was performed on a ChemiDoc™ Touch Imaging System (Bio-Rad Laboratories).

### 4.5. Cytokine Detection Assays

HDFs (10^4^/well) were seeded and grown overnight in a 96-well plate (BD Biosciences). The cells were treated for 24 h with a mixture of rhTNF-α and different concentrations of DMSO (0.1–100%) (preincubated for 1 h). The secretion level of IL-8 was assessed with the Human IL-8 Uncoated ELISA Kit (eBioscience, Inc., San Diego, CA, USA), while that of IL-6 was measured using the Human IL-6 ELISA MAX Deluxe Kit (BioLegend, San Diego, CA, USA). The absorbance in the plate wells was read on a microplate spectrophotometer system (Molecular Devices).

### 4.6. Dissociation of a TNF-α Oligomer

Different concentrations of DMSO were incubated for 1 h at 37 °C with 100 ng of recombinant TNF-α, followed by cross-linking with 4.8 mM BS3 (Thermo Fisher Scientific, Inc.) for 30 min at room temperature. Next, the reaction was stopped with a ^1^/_10_ volume of 1 M Tris-HCl (pH 7.5). Proteins in the samples were separated by SDS-PAGE and processed for Western blotting analysis with an anti-TNF-α antibody (Cell Signaling Technology Inc., Baverly, MA, USA).

### 4.7. MD Simulations

The topology of DMSO molecules was obtained from the PRODRG server [74]. MD simulations were performed in the GROMACS software with the GROMOS96-54A7 force field. Different numbers of DMSO molecules were docked into the hydrophobic cavity of the TNF homodimer [PDB ID: 2AZ5 [37]] using the MOE software [75]. The London dG scoring function was employed to rank the best poses. The DMSO–TNF complexes were solvated with SPC216 water molecules by implementing a periodic boundary condition. The simulation systems were neutralized with an appropriate amount of counterions. Steepest-descent minimization was performed until the maximum force reached 1000 kJ mol^−1^ nm^−1^. During position-restraint simulations, the temperature was equilibrated with the V-rescale scheme for 100 ps, and the pressure equilibration was performed via the Parrinello–Rahman algorithm at 1 bar for 100 ps. Next, a 30 ns production run was carried out without backbone restraints, and all bonds involving hydrogen atoms were constrained by a linear constraint solver algorithm. The trajectory data were saved every 10 ps, and the time stamp of 0.002 ps was set for the simulation. Data analysis and visualization were carried out using the VMD software [76] and built-in GROMACS tools.

### 4.8. Statistical Analysis

All in vitro data analyses were performed by the two-tailed paired Student’s *t*-test in Microsoft Excel 2016. All data are presented as mean ± SEM. *p* values < 0.05 were assumed to indicate statistical significance.

## 5. Conclusions

Drug-like compounds are usually dissolved in the polar organic solvent DMSO for the initial screening process. DMSO by itself can affect the target molecule. For example, TNF-α is an inflammatory cytokine that is involved in multiple diseases such as rheumatoid arthritis, Crohn’s disease, and psoriasis. The presence of DMSO as a solvent interrupts the functioning of TNF-α at the molecular level in terms of activation of associated signaling pathway components, induction of caspase-mediated cell death, and induction of secretion of ultimate inflammatory cytokines, such as IL-8 and IL-6. This inhibition is directly linked to the concentration of DMSO present in the solution. In some cells, sensitization by transcriptional arrest is necessary for the cell death pathway to prevail over cell survival/proliferative pathways. This sensitization process depends on the cell type and on the timing of treatments. According to our results, a proper time gap between act-D and TNF-α treatments, a suitable concentration of added DMSO, and/or a suitable alternative solvent for drug-like compounds must be considered for the screening of drug-like compounds.

## Supplementary Materials

The following are available online at https://www.mdpi.com/1422-0067/21/24/9450/s1.

## Abbreviations

act-D	actinomycin D
DMSO	dimethyl sulfoxide
HDFs	human dermal fibroblasts
hIL-6	human interleukin 6
hIL-8	human interleukin 8
MAPK	mitogen-activated protein kinase
MD	molecular dynamics
MTT	1-(4,5-dimethylthiazol-2-yl)-3,5-diphenylformazan
p-	phospho-
rhTNF-α	recombinant human TNF-α
rmTNF-α	recombinant murine TNF-α
TNF	tumor necrosis factor

## References

[1] Aggarwal B.B., Gupta S.C., Kim J.H. (2012). Historical perspectives on tumor necrosis factor and its superfamily: 25 years later, a golden journey. Blood.

[2] Beutler B., Milsark I.W., Cerami A.C. (1985). Passive immunization against cachectin/tumor necrosis factor protects mice from lethal effect of endotoxin. Science.

[3] Victor F.C., Gottlieb A.B. (2002). TNF-alpha and apoptosis: Implications for the pathogenesis and treatment of psoriasis. J. Drugs Derm..

[4] Brynskov J., Foegh P., Pedersen G., Ellervik C., Kirkegaard T., Bingham A., Saermark T. (2002). Tumour necrosis factor alpha converting enzyme (TACE) activity in the colonic mucosa of patients with inflammatory bowel disease. Gut.

[5] Locksley R.M., Killeen N., Lenardo M.J. (2001). The TNF and TNF receptor superfamilies: Integrating mammalian biology. Cell.

[6] Dowlati Y., Herrmann N., Swardfager W., Liu H., Sham L., Reim E.K., Lanctot K.L. (2010). A meta-analysis of cytokines in major depression. Biol. Psychiatry.

[7] Swardfager W., Lanctot K., Rothenburg L., Wong A., Cappell J., Herrmann N. (2010). A meta-analysis of cytokines in Alzheimer’s disease. Biol. Psychiatry.

[8] Monaco C., Nanchahal J., Taylor P., Feldmann M. (2015). Anti-TNF therapy: Past, present and future. Int. Immunol..

[9] Eck M.J., Sprang S.R. (1989). The structure of tumor necrosis factor-alpha at 2.6 A resolution. Implications for receptor binding. J. Biol. Chem..

[10] Marušič J., Podlipnik Č., Jevševar S., Kuzman D., Vesnaver G., Lah J. (2012). Recognition of Human Tumor Necrosis Factor α (TNF-α) by Therapeutic Antibody Fragment Energetics and Structural Features. J. Biol. Chem..

[11] Pennica D., Lam V.T., Weber R.F., Kohr W.J., Basa L.J., Spellman M.W., Ashkenazi A., Shire S.J., Goeddel D.V. (1993). Biochemical characterization of the extracellular domain of the 75-kilodalton tumor necrosis factor receptor. Biochemistry.

[12] Loetscher H., Gentz R., Zulauf M., Lustig A., Tabuchi H., Schlaeger E., Brockhaus M., Gallati H., Manneberg M., Lesslauer W. (1991). Recombinant 55-kDa tumor necrosis factor (TNF) receptor. Stoichiometry of binding to TNF alpha and TNF beta and inhibition of TNF activity. J. Biol. Chem..

[13] Roy U. (2017). Structural modeling of tumor necrosis factor: A protein of immunological importance. Biotechnol. Appl. Biochem..

[14] Roy U. (2019). 3D Modeling of Tumor Necrosis Factor Receptor and Tumor Necrosis Factor-bound Receptor Systems. Mol. Inform..

[15] Narhi L.O., Arakawa T. (1987). Dissociation of recombinant tumor necrosis factor-α studied by gel permeation chromatography. Biochem. Biophys. Res. Commun..

[16] Corti A., Fassina G., Marcucci F., Barbanti E., Cassani G. (1992). Oligomeric tumour necrosis factor α slowly converts into inactive forms at bioactive levels. Biochem. J..

[17] Ameloot P., Declercq W., Fiers W., Vandenabeele P., Brouckaert P. (2001). Heterotrimers formed by tumor necrosis factors of different species or muteins. J. Biol. Chem..

[18] Kunitani M.G., Cunico R.L., Staats S.J. (1988). Reversible subunit dissociation of tumor necrosis factor during hydrophobic interaction chromatography. J. Chromatogr. A.

[19] Beil E.J., Heavner G.A., Wu S.J., Nemeth J.F. (2012). Probing the solution structure of tumor necrosis factor-α homotrimer and heterotrimer after complex perturbation using electrospray ionization mass spectrometry. J. Mol. Recognit..

[20] Verwer P., Leusen F.J. (1998). Computer simulation to predict possible crystal polymorphs. Rev. Comput. Chem..

[21] Davies D.T. (1992). Aromatic Heterocyclic Chemistry.

[22] Marcus Y. (1998). The Properties of Solvents.

[23] Reichardt C., Welton T. (2011). Solvents and Solvent Effects in Organic Chemistry.

[24] Kennedy T. (1997). Managing the drug discovery/development interface. Drug Discov. Today.

[25] Alsenz J., Kansy M. (2007). High throughput solubility measurement in drug discovery and development. Adv. Drug Deliv. Rev..

[26] Di L., Fish P.V., Mano T. (2012). Bridging solubility between drug discovery and development. Drug Discov. Today.

[27] MacGregor W.S. (1967). The chemical and physical properties of DMSO. Ann. N. Y. Acad. Sci..

[28] Brown J. (1971). A double-blind clinical study—DMSO for acute injuries and inflammations compared to accepted standard therapy. Curr. Ther. Res. Clin. Exp..

[29] Régnier J., Richard J. (1998). Lack of developmental toxicity in rats treated with dimethylsulfoxide (DMSO). Toxicologist.

[30] O’Connell J., Porter J., Kroeplien B., Norman T., Rapecki S., Davis R., McMillan D., Arakaki T., Burgin A., Fox Iii D. (2019). Small molecules that inhibit TNF signalling by stabilising an asymmetric form of the trimer. Nat. Commun..

[31] Niwayama S., Turk B.E., Liu J.O. (1996). Potent inhibition of tumor necrosis factor-α production by tetrafluorothalidomide and tetrafluorophthalimides. J. Med. Chem..

[32] Sampaio E.P., Sarno E.N., Galilly R., Cohn Z.A., Kaplan G. (1991). Thalidomide selectively inhibits tumor necrosis factor alpha production by stimulated human monocytes. J. Exp. Med..

[33] Jackson R.W., Gelinas R., Baughman T.A., Cox T., Howbert J.J., Kucera K.A., Latham J.A., Ramsdell F., Singh D., Darwish I.S. (2002). Benzobicyclooctanes as novel inhibitors of TNF-α signaling. Bioorganic Med. Chem. Lett..

[34] Luzi S., Kondo Y., Bernard E., Stadler L.K., Vaysburd M., Winter G., Holliger P. (2015). Subunit disassembly and inhibition of TNFα by a semi-synthetic bicyclic peptide. Protein Eng. Des. Sel..

[35] Papaneophytou C., Alexiou P., Papakyriakou A., Ntougkos E., Tsiliouka K., Maranti A., Liepouri F., Strongilos A., Mettou A., Couladouros E. (2015). Synthesis and biological evaluation of potential small molecule inhibitors of tumor necrosis factor. MedChemComm.

[36] Hu Z., Qin J., Zhang H., Wang D., Hua Y., Ding J., Shan L., Jin H., Zhang J., Zhang W. (2012). Japonicone A antagonizes the activity of TNF-α by directly targeting this cytokine and selectively disrupting its interaction with TNF receptor-1. Biochem. Pharmacol..

[37] He M.M., Smith A.S., Oslob J.D., Flanagan W.M., Braisted A.C., Whitty A., Cancilla M.T., Wang J., Lugovskoy A.A., Yoburn J.C. (2005). Small-molecule inhibition of TNF-alpha. Science.

[38] Douni E., Kollias G. (1998). A critical role of the p75 tumor necrosis factor receptor (p75TNF-R) in organ inflammation independent of TNF, lymphotoxin alpha, or the p55TNF-R. J. Exp. Med..

[39] Ting A.T., Bertrand M.J.M. (2016). More to Life than NF-kappaB in TNFR1 Signaling. Trends Immunol..

[40] Annibaldi A., Meier P. (2018). Checkpoints in TNF-Induced Cell Death: Implications in Inflammation and Cancer. Trends Mol. Med..

[41] Wajant H., Pfizenmaier K., Scheurich P. (2003). Tumor necrosis factor signaling. Cell Death Differ..

[42] De Abreu Costa L., Henrique Fernandes Ottoni M., Dos Santos M.G., Meireles A.B., Gomes de Almeida V., de Fátima Pereira W., Alves de Avelar-Freitas B., Eustáquio Alvim Brito-Melo G. (2017). Dimethyl sulfoxide (DMSO) decreases cell proliferation and TNF-α, IFN-γ, and IL-2 cytokines production in cultures of peripheral blood lymphocytes. Molecules.

[43] Da Violante G., Zerrouk N., Richard I., Provot G., Chaumeil J.C., Arnaud P. (2002). Evaluation of the cytotoxicity effect of dimethyl sulfoxide (DMSO) on Caco2/TC7 colon tumor cell cultures. Biol. Pharm. Bull..

[44] Ahn H., Kim J., Jeung E.-B., Lee G.-S. (2014). Dimethyl sulfoxide inhibits NLRP3 inflammasome activation. Immunobiology.

[45] Tselepis C., Perry I., Dawson C., Hardy R., Darnton S.J., McConkey C., Stuart R.C., Wright N., Harrison R., Jankowski J.A.Z. (2002). Tumour necrosis factor-α in Barrett’s oesophagus: A potential novel mechanism of action. Oncogene.

[46] Humblet C., Greimers R., Delvenne P., Deman J., Boniver J., Defresne M.P. (1996). Prevention of murine radiogenic thymic lymphomas by tumor necrosis factor or by marrow grafting. JNCI J. Natl. Cancer Inst..

[47] Elinav E., Nowarski R., Thaiss C.A., Hu B., Jin C., Flavell R.A. (2013). Inflammation-induced cancer: Crosstalk between tumours, immune cells and microorganisms. Nat. Rev. Cancer.

[48] Brenner D., Blaser H., Mak T.W. (2015). Regulation of tumour necrosis factor signalling: Live or let die. Nat. Rev. Immunol..

[49] Blevitt J.M., Hack M.D., Herman K.L., Jackson P.F., Krawczuk P.J., Lebsack A.D., Liu A.X., Mirzadegan T., Nelen M.I., Patrick A.N. (2017). Structural Basis of Small-Molecule Aggregate Induced Inhibition of a Protein-Protein Interaction. J. Med. Chem..

[50] Chen S., Feng Z., Wang Y., Ma S., Hu Z., Yang P., Chai Y., Xie X. (2017). Discovery of novel ligands for TNF-α and TNF receptor-1 through structure-based virtual screening and biological assay. J. Chem. Inf. Model..

[51] Szmant H.H. (1975). Physical properties of dimethyl sulfoxide and its function in biological systems. Ann. N. Y. Acad. Sci..

[52] Sankpal U.T., Abdelrahim M., Connelly S.F., Lee C.M., Madero-Visbal R., Colon J., Smith J., Safe S., Maliakal P., Basha R. (2012). Small molecule tolfenamic acid inhibits PC-3 cell proliferation and invasion in vitro, and tumor growth in orthotopic mouse model for prostate cancer. Prostate.

[53] Modesitt S.C., Parsons S.J. (2010). In vitro and in vivo histone deacetylase inhibitor therapy with vorinostat and paclitaxel in ovarian cancer models: Does timing matter?. Gynecol. Oncol..

[54] Li S.-Y., Lo A.C. (2010). Lutein protects RGC-5 cells against hypoxia and oxidative stress. Int. J. Mol. Sci..

[55] Julien C., Marcouiller F., Bretteville A., El Khoury N.B., Baillargeon J., Hébert S.S., Planel E. (2012). Dimethyl sulfoxide induces both direct and indirect tau hyperphosphorylation. PLoS ONE.

[56] Hanslick J.L., Lau K., Noguchi K.K., Olney J.W., Zorumski C.F., Mennerick S., Farber N.B. (2009). Dimethyl sulfoxide (DMSO) produces widespread apoptosis in the developing central nervous system. Neurobiol. Dis..

[57] Feldmann M., Maini R.N. (2001). Anti-TNFα therapy of rheumatoid arthritis: What have we learned?. Ann. Rev. Immunol..

[58] Bradley J. (2008). TNF-mediated inflammatory disease. J. Pathol..

[59] Pennica D., Nedwin G.E., Hayflick J.S., Seeburg P.H., Derynck R., Palladino M.A., Kohr W.J., Aggarwal B.B., Goeddel D.V. (1984). Human tumour necrosis factor: Precursor structure, expression and homology to lymphotoxin. Nature.

[60] Kriegler M., Perez C., DeFay K., Albert I., Lu S. (1988). A novel form of TNF/cachectin is a cell surface cytotoxic transmembrane protein: Ramifications for the complex physiology of TNF. Cell.

[61] Luettig B., Decker T., Lohmann-Matthes M. (1989). Evidence for the existence of two forms of membrane tumor necrosis factor: An integral protein and a molecule attached to its receptor. J. Immunol..

[62] Moss M.L., Jin S.-L.C., Milla M.E., Burkhart W., Carter H.L., Chen W.-J., Clay W.C., Didsbury J.R., Hassler D., Hoffman C.R. (1997). Cloning of a disintegrin metalloproteinase that processes precursor tumour-necrosis factor-α. Nature.

[63] Vandenabeele P., Declercq W., Beyaert R., Fiers W. (1995). Two tumour necrosis factor receptors: Structure and function. Trends Cell Biol..

[64] Tang P., Hung M.-C., Klostergaard J. (1996). Human pro-tumor necrosis factor is a homotrimer. Biochemistry.

[65] Papaneophytou C.P., Mettou A.K., Rinotas V., Douni E., Kontopidis G.A. (2013). Solvent selection for insoluble ligands, a challenge for biological assay development: A TNF-α/SPD304 study. ACS Med. Chem. Lett..

[66] Micheau O., Tschopp J. (2003). Induction of TNF receptor I-mediated apoptosis via two sequential signaling complexes. Cell.

[67] Beg A.A., Baltimore D. (1996). An essential role for NF-κB in preventing TNF-α-induced cell death. Science.

[68] Wang C.-Y., Mayo M.W., Baldwin A.S. (1996). TNF-and cancer therapy-induced apoptosis: Potentiation by inhibition of NF-κB. Science.

[69] Martin S., Lennon S., Bonham A., Cotter T. (1990). Induction of apoptosis (programmed cell death) in human leukemic HL-60 cells by inhibition of RNA or protein synthesis. J. Immunol..

[70] Fulda S., Meyer E., Debatin K.-M. (2000). Metabolic inhibitors sensitize for CD95 (APO-1/Fas)-induced apoptosis by down-regulating Fas-associated death domain-like interleukin 1-converting enzyme inhibitory protein expression. Cancer Res..

[71] Gozzelino R., Sole C., Llecha N., Segura M.F., Moubarak R.S., Iglesias-Guimarais V., Perez-Garcia M.J., Reix S., Zhang J., Badiola N. (2008). BCL-X L regulates TNF-α-mediated cell death independently of NF-κB, FLIP and IAPs. Cell Res..

[72] Leist M., Gantner F., Bohlinger I., Germann P.G., Tiegs G., Wendel A. (1994). Murine hepatocyte apoptosis induced in vitro and in vivo by TNF-alpha requires transcriptional arrest. J. Immunol..

[73] Ma L., Gong H., Zhu H., Ji Q., Su P., Liu P., Cao S., Yao J., Jiang L., Han M. (2014). A novel small-molecule tumor necrosis factor α inhibitor attenuates inflammation in a hepatitis mouse model. J. Biol. Chem..

[74] Schuttelkopf A.W., van Aalten D.M. (2004). PRODRG: A tool for high-throughput crystallography of protein-ligand complexes. Acta Cryst. D Biol. Cryst..

[75] MOE (2017). Molecular Operating Environment (MOE), 2013.08.

[76] Humphrey W., Dalke A., Schulten K. (1996). VMD: Visual molecular dynamics. J. Mol. Graph..

